# Expression of the Tau Protein and Amyloid Protein Precursor Processing Genes in the CA3 Area of the Hippocampus in the Ischemic Model of Alzheimer’s Disease in the Rat

**DOI:** 10.1007/s12035-019-01799-z

**Published:** 2019-11-12

**Authors:** Ryszard Pluta, Marzena Ułamek-Kozioł, Janusz Kocki, Jacek Bogucki, Sławomir Januszewski, Anna Bogucka-Kocka, Stanisław J. Czuczwar

**Affiliations:** 1 Laboratory of Ischemic and Neurodegenerative Brain Research, Mossakowski Medical Research Centre, Polish Academy of Sciences, Pawińskiego 5 Str, 02-106 Warsaw, Poland; 2 First Department of Neurology, Institute of Psychiatry and Neurology, Warsaw, Poland; 3 Department of Clinical Genetics, Medical University of Lublin, Lublin, Poland; 4 Department of Biology and Genetics, Medical University of Lublin, Lublin, Poland; 5 Department of Pathophysiology, Medical University of Lublin, Lublin, Poland

**Keywords:** Brain ischemia, Alzheimer’s disease, Amyloid protein precursor, α-Secretase, β-Secretase, Presenilin 1 and 2, Tau protein

## Abstract

Understanding the mechanisms underlying the selective susceptibility to ischemia of the CA3 region is very important to explain the neuropathology of memory loss after brain ischemia. We used a rat model to study changes in gene expression of the amyloid protein precursor and its cleaving enzymes and tau protein in the hippocampal CA3 sector, after transient 10-min global brain ischemia with survival times of 2, 7, and 30 days. The expression of the α-secretase gene was below control values at all times studied. But, the expression of the β-secretase gene was below the control values at 2–7 days after ischemia and the maximal increase in its expression was observed on day 30. Expression of the presenilin 1 gene was significantly elevated above the control values at 2–7 days after ischemia and decreased below the control values at day 30. Expression of the presenilin 2 gene showed an opposite trend to the expression of presenilin 1. Expression of the amyloid protein precursor gene after ischemia was at all times above the control values with a huge significant overexpression on day 7. Additionally, the expression of the tau protein gene was below the control values 2 days after ischemia, but the significant increase in its expression was observed on days 7–30. Data show that brain ischemia activates neuronal changes and death in the CA3 region of the hippocampus in a manner dependent on amyloid and tau protein, thus determining a new and important way to regulate the survival and/or death of ischemic neurons.

## Introduction

The hippocampus is an important part of the brain due to its anatomical structure and physiological functions, especially in the area related to learning and memory under normal conditions and dementia in Alzheimer’s disease and brain injury after ischemia [1–7]. The hippocampus plays an important role in the consolidation of information from short-term memory to long-term memory and spatial orientation, and this important transmission is disturbed inter alia in Alzheimer’s disease [3, 4]. In addition, various areas of pyramidal neurons in the hippocampus also show a rather specialized response to pathological factors, as exemplified by similar neurodegenerative changes in Alzheimer’s disease and cerebral ischemia [5, 8–10].

Alzheimer’s disease is a complex, chronic neurodegenerative disorder that leads to the death of neurons primarily in the hippocampus, brain atrophy and dementia [5, 10]. It is evident that the first structure affected by Alzheimer’s disease is the hippocampus, especially its CA1 region, where a significant decrease in neuron density is observed compared with the less significant one in the CA3 region [10]. Transient experimental brain ischemia also causes early death of pyramidal neurons in the CA1 region, while neurons in the nearby CA3 region remain relatively spared [11–13]. In our previous studies, we found gene expression changes associated with Alzheimer’s disease in the hippocampal CA1 region, such as β-secretase, presenilin 1 and 2, amyloid protein precursor, and tau protein during 2–7 days after brain ischemia [8, 9, 14, 15]. In addition, morphological observations have shown an increased number of disrupted pyramidal neurons in the CA3 region with acute and chronic lesions [11, 12] during 2 years of survival following brain ischemia [9] as one of the characteristic features for the development of dementia in Alzheimer’s disease [10]. It seems that changes in pyramidal neurons, in particular in the hippocampus, such as the CA3 region, may play a significant role in memory deficits that herald the onset of Alzheimer’s disease [5] and the development of Alzheimer’s disease dementia after brain injury due to ischemia-reperfusion [2, 4].

The hippocampal regions, including the CA3 region, are currently the main focus of research on gene expression [14, 15] due to its involvement in learning and memory and participation in the development of similar neurodegenerative changes in dementia resulting from Alzheimer’s disease or brain ischemia [2–5, 10, 16]. Mechanisms affecting different susceptibility to ischemia of pyramidal neurons in the hippocampus have recently been extensively studied in the CA1 region, while in the CA3 area, there is no new data in this regard. To date, only one experimental article in the literature has investigated differences in gene expression between the CA1 and CA3 areas of the hippocampus after focal brain ischemia with hypoxia in mice [17]. The most surprising result of the above study is that the strong transcriptional stimulus like ischemia with hypoxia leads to a significant reduction in pre-existing cell-specific gene expression patterns instead of generating new or increasing existing differences [17]. On the other hand, examination of the brains of very old men with rare senile plaques and neurofibrillary tangles showed increased expression of the amyloid protein precursor and α-synuclein in CA3 area neurons compared with CA1 [18]. Additionally, it was noted that neurodegeneration of the hippocampal CA1 region after experimental cerebral ischemia was somewhat dependent on amyloid and tau protein [14, 15]. Thus, studies on differences in the modulation of the amyloid and tau protein gene may provide key functional elements of the molecular landscape during neurodegeneration of CA1 and CA3 in the brain after cerebral ischemia and Alzheimer’s disease.

In the last years, more and more evidence points to the role of brain ischemia in the pathogenesis of Alzheimer’s disease [1, 6, 19–23]. The above suggestion is confirmed by epidemiological observations indicating that ischemic stroke is a factor contributing to the development of Alzheimer’s disease and vice versa [20]. Interestingly, this is also confirmed by similar neuropathological features observed after experimental ischemic brain damage as well as in Alzheimer’s disease [5, 6, 9, 22, 23]. However, the mechanism by which brain ischemia can lead to the development of full-blown Alzheimer’s disease remains still unclear. Understanding the genomic and proteomic effects of ischemia on the pathogenesis of Alzheimer’s disease will be of great importance in explaining the etiology of this debilitating disorder. Perhaps the ischemic regional selectivity of neuronal death in the CA1 and CA3 areas may be an important factor in understanding the mechanisms associated with the development of Alzheimer’s disease. Understanding the mechanisms underlying the selective vulnerability to ischemia of the CA3 area is very important to explain the neuropathology of memory loss after brain ischemia with reperfusion. This report is part of a continuous series of studies that focus on quantitative analysis, using RT-PCR protocol, selected genes associated with Alzheimer’s disease in various rat brain structures, such as α-secretase, β-secretase, presenilin 1 and 2, amyloid protein precursor, and tau protein to assess the selective susceptibility to ischemia of pyramidal neurons in the CA3 region of the hippocampus during development of neurodegeneration and dementia after ischemia.

## Materials and Methods

In female Wistar rats (*n* = 30, 2 months old, weight 160–180 g), 10-min brain ischemia was induced by cardiac arrest [24]. Briefly, rats were anesthetized with 2.0% isoflurane carried by O_2_. Shortly before starting the cardiac arrest procedure, anesthesia was discontinued. A special blunt L-shaped hook made of a straight stiff steel needle was inserted through the right parasternal line through the third intercostal space into the chest cavity. At this stage, the hook was positioned parallel to the right parasternal line, and the closing part of the hook was in a vertical position inside the chest. Then, the shaft of the hook was moved to a vertical position. At that time, the short end of the hook was completely inside the chest and plural cavity. Then, the hook was gently pressed down to the vertebral column until slight resistance was found. Then, the hook was tilted slightly 10–20° in the tail direction. The hook was next rotated counterclockwise approximately 135–140° under the inferior vena cava. In this position, the closing part of the hook was placed under the heart vessel bundle. In the last stage, the hook was pulled up to the sternum, which caused the heart vessel bundle to be compressed by the sternum. The end of the closing part of the hook in the above arrangement was in the left parasternal line in the second intercostal space. To prevent chest movement and ensure complete vascular occlusion, the index and middle finger pressure was applied to the sternum, resulting in complete hemostasis, followed by cardiac arrest. The trachea was not closed during the whole procedure. After 3.5 min, cardiac arrest was noted and then the hook was removed from the chest in reverse order and the rats remained in this state until resuscitation began. The resuscitation procedure consisted of external heart massage and artificial ventilation until spontaneous heart activity was restored and breathing appeared. At this time, air was pumped through a polyethylene tube inserted into the trachea, which was connected to a respirator. External heart massage was performed with the index and middle finger, rapidly hitting the chest at the level of the fourth intercostal area with a frequency of 150–240/min continuously. The ratio of strokes to the frequency of ventilation was 6:1 or 8:1. The 10 min of cerebral ischemia consisted of 3.5 min of heart vessel bundle compression by the hook and 6.5 min after removing the hook from the chest [24].

The animals were kept in pairs in cages in a room at a controlled temperature of 23 ± 1 °C, humidity 55 ± 5%, and a cycle of 12-h light/dark. They had unlimited access to commercial granular laboratory chow and tap water. The experiments were carried out in a light phase and the animals were treated according to the NIH Guide on the care and use of laboratory animals and a directive of the Council of the European Communities 142. The Local Ethical Committee approved all planned experimental procedures. After brain ischemia, the survival time of the animals was 2 (*n* = 14), 7 (*n* = 8), and 30 (*n* = 8) days. Sham-operated rats (*n* = 30) with a survival time of 2 days (*n* = 14), 7 days (*n* = 8), and 30 days (*n* = 8) were subjected to the same experimental procedures without global cerebral ischemia and served as appropriate control groups.

Before the hippocampal sample from the CA3 region was taken, the brains were perfused with cold 0.9% NaCl through the left ventricle to rinse the blood vessels from the blood. Then, the brains were removed from the skulls and transferred to an ice-cooled Petri dish. Samples were collected from the CA3 region of the ischemic and control hippocampus with a narrow scalpel of approximately 1 mm^3^ on both sides and immediately placed in the RNAlater solution (Life Technologies, USA) [14].

The isolation of total cellular RNA was made using the method developed by Chomczyński and Sacchi [25]. The NanoDrop 2000 spectrophotometer (Thermo Scientific, USA) was used to assess the quality and quantity of RNA [14, 15, 26–29]. The isolated RNA was stored in 80% ethanol at – 20 °C for further analyses [14, 15, 26–29]. In further studies, 1 μg of total RNA was reverse transcribed into cDNA using a high-capacity cDNA kit for reverse transcription according to the manufacturer’s instructions (Applied Biosystems, USA). The cDNA synthesis was performed on Veriti Dx (Applied Biosystems, USA) under the following conditions: stage I 25 °C, 10 min; stage II 37 °C, 120 min; stage III 85 °C, 5 min; stage IV 4 °C. The cDNA, obtained by this procedure, was amplified by real-time gene expression analysis (qPCR) on a 7900HT Real-Time Fast System (Applied Biosystems, USA) with the Master Mix SYBR-green PCR power mix reagent, using the manufacturer’s SDS software [14]. The amplification protocol included the following cycles: initial denaturation 95 °C, 10 min, and 40 cycles, each at two different temperatures 95 °C, 15 s, and 60 °C, 1 min. The monitoring and calculation of the number of DNA copies was carried out in the 7900HT Real-Time Fast System (Applied Biosystems, USA) in each amplification cycle. The number of cycles of PCR at which the fluorescence level exceeded the specific relative expression of the threshold cycle (CT) was applied to the research software (Applied Biosystems, USA) to calculate the number of DNA molecules present in the mixture at the beginning of the reaction. Normalization was achieved against the endogenous control gene (Rpl13a) [30], and the relative amount (RQ) of the gene expression was analyzed based on the ΔCT method and the results were calculated as RQ = 2^−ΔΔCT^ [14, 31]. The RQ values were finally analyzed after their logarithmic conversion to the RQ logarithm (LogRQ) [14]. LogRQ = 0 indicates that gene expression in control and ischemic samples is not different. LogRQ < 0 means that we have reduced gene expression in the ischemic sample, while LogRQ > 0 indicates increased gene expression in the ischemic sample compared with the control one.

Statistical evaluation of the results was carried out using the Statistica v. 12 software with the help of non-parametric Kruskal-Wallis test with the “z” test—multiple analyses of differences between groups. Data are presented as means ± SD. Statistical significance was adopted at *p* ≤ 0.05.

## Results

### Expression of the α-Secretase Gene after Ischemia

**Fig. 1:**
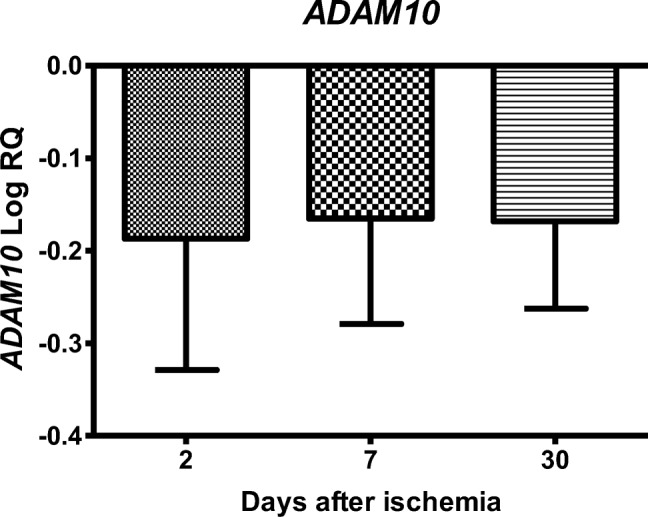
The mean gene expression levels of α-secretase (*ADAM10*) in the hippocampus CA3 area 2 (*n* = 14), 7 (*n* = 8), and 30 (*n* = 8) days after 10-min global brain ischemia. Marked SD, standard deviation. No statistical significance at all times after ischemia (Kruskal-Wallis test)

In the CA3 region of the hippocampus, the expression of the α-secretase (*ADAM10*) gene after 10-min brain ischemia with a survival of 2, 7, and 30 days was below the control values. On the second day after ischemia, the minimum was − 0.524-fold change and maximum − 0.004-fold change with median − 0.139-fold change. On the seventh day after ischemia, the minimum was − 0.344-fold change and maximum − 0.044-fold change with median − 0.136-fold change. On the thirtieth day after ischemia, the minimum was − 0.250-fold change and maximum − 0.009-fold change with median − 0.197-fold change. Figure 1 illustrates changes in the mean level of expression of the α-secretase gene. The changes were not statistically significant at all the times after ischemia (Fig. 1).

### Expression of the β-Secretase Gene after Ischemia

**Fig. 2:**
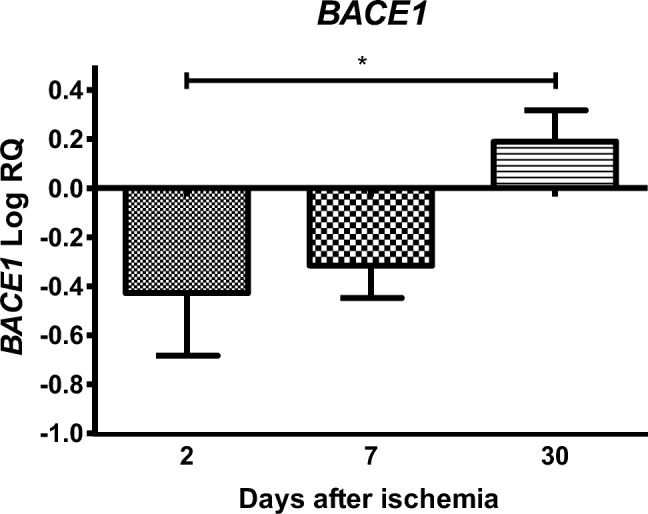
The mean gene expression levels of β-secretase (*BACE1*) in the hippocampus CA3 area in rats 2 (*n* = 14), 7 (*n* = 8), and 30 (*n* = 8) days after 10-min global brain ischemia. Marked SD, standard deviation. Indicated statistically significant differences in levels of gene expression between 2 and 30 (*z* = 3.368, *p* = 0.0022) days after ischemia (Kruskal-Wallis test). *p ≤ 0.01

In the CA3 region of the hippocampus, the expression of the β-secretase gene (*BACE1*) after ischemia with 2- and 7-day survival was lower than the control values, but on day 30, it was higher than the control values. On the second day after ischemia, the minimum was − 1.086-fold change and maximum − 0.001-fold change with median − 0.382-fold change. On the seventh day after ischemia, the minimum was − 0.457-fold change and maximum − 0.136-fold change with median − 0.334-fold change. On the thirtieth day after ischemia, the minimum was 0.043-fold change and maximum 0.336-fold change with median 0.189-fold change. Figure 2 illustrates changes in the mean level of expression of the β-secretase gene. The changes were statistically significant between 2 and 30 days after ischemia (Fig. 2).

### Expression of the Presenilin 1 Gene after Ischemia

**Fig. 3:**
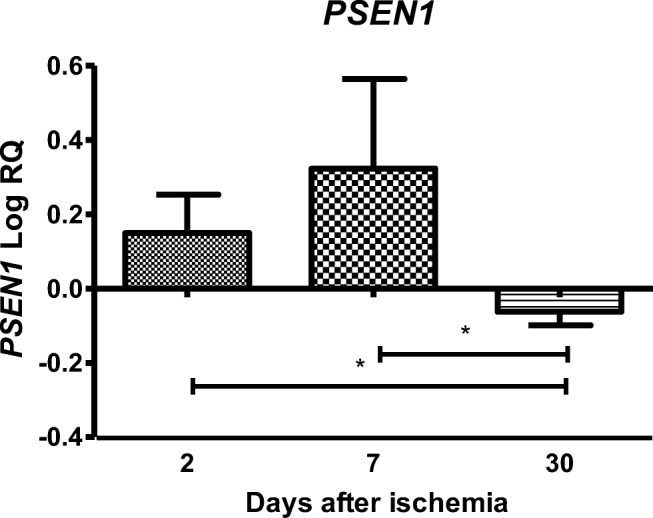
The mean gene expression levels of presenilin 1 (*PSEN1*) in the hippocampus CA3 area in rats 2 (*n* = 14), 7 (*n* = 8), and 30 (*n* = 8) days after 10-min global brain ischemia. Marked SD, standard deviation. Indicated statistically significant differences in levels of gene expression between 2 and 30 (*z* = 3.825, *p* = 0.0003) and between 7 and 30 (*z* = 3.920, *p* = 0.0002) days after ischemia (Kruskal-Wallis test). *p ≤ 0.001

In the studied region, the expression of the presenilin 1 gene (*PSEN1*) after ischemia with a survival of 2 and 7 days was higher than the control values and lower than the control values at day 30. On the second day after ischemia, the minimum was 0.004-fold change and maximum 0.430-fold change with median 0.131-fold change. On the seventh day after ischemia, the minimum was 0.029-fold change and maximum 0.584-fold change with median 0.356-fold change. On the thirtieth day after ischemia, the minimum was − 0.105-fold change and maximum − 0.019-fold change with median − 0.062-fold change. Figure 3 illustrates changes in the mean level of expression of the presenilin 1 gene. The changes were statistically significant between 2 and 30 days and between 7 and 30 days after ischemia (Fig. 3).

### Expression of the Presenilin 2 Gene after Ischemia

**Fig. 4:**
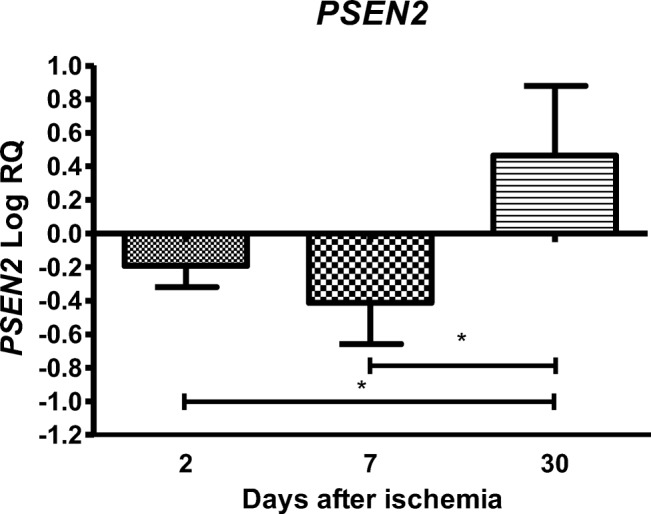
The mean gene expression levels of presenilin 2 (*PSEN2*) in the hippocampus CA3 area in rats 2 (*n* = 14), 7 (*n* = 8), and 30 (*n* = 8) days after 10-min global brain ischemia. Marked SD, standard deviation. Indicated statistically significant differences in levels of gene expression between 2 and 30 (*z* = 4.257, *p* = 0.00006) and between 7 and 30 (*z* = 4.550, *p* = 0.00001) days after ischemia (Kruskal-Wallis test). *p ≤ 0.0001

In the CA3 area, the expression of the presenilin 2 gene (*PSEN2*) after ischemia-reperfusion injury with a survival of 2 and 7 days was lower than the control values and higher than the control values at day 30. On the second day after ischemia, the minimum was − 0.498-fold change and maximum − 0.003-fold change with median − 0.177-fold change. On the seventh day after ischemia, the minimum was − 0.695-fold change and maximum − 0.034-fold change with median − 0.465-fold change. On the thirtieth day after ischemia, the minimum was 0.035-fold change and maximum 1.135-fold change with median 0.358-fold change. Figure 4 illustrates changes in the mean level of expression of the presenilin 2 gene. The changes were statistically significant between 2 and 30 days and between 7 and 30 days after ischemia (Fig. 4).

### Expression of the Amyloid Protein Precursor Gene after Ischemia

**Fig. 5:**
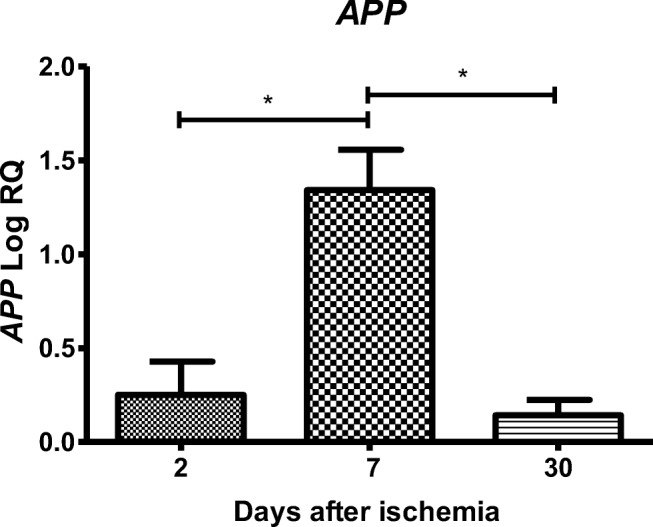
The mean gene expression levels of amyloid protein precursor (*APP*) in the hippocampus CA3 area in rats 2 (*n* = 14), 7 (*n* = 8), and 30 (*n* = 8) days after 10-min global brain ischemia. Marked SD, standard deviation. Indicated statistically significant differences in levels of gene expression between 2 and 7 (*z* = 3.498, *p* = 0.0014) and between 7 and 30 (*z* = 3.467, *p* = 0.0015) days after ischemia (Kruskal-Wallis test). *p ≤ 0.01

After ischemia in the CA3 region of the hippocampus, the expression of the amyloid protein precursor gene during 2, 7, and 30 days of survival was above the control values. On the second day after ischemia, the minimum was 0.029-fold change and maximum 0.698-fold change with median 0.213-fold change. On the seventh day after ischemia, the minimum was 1.164-fold change and maximum 1.718-fold change with median 1.279-fold change. On the thirtieth day after ischemia, the minimum was 0.055-fold change and maximum 0.223-fold change with median 0.156-fold change. Figure 5 illustrates changes in the mean level of expression of the amyloid protein precursor gene. The changes were statistically significant between 2 and 7 days and between 7 and 30 days after ischemia (Fig. 5).

### Expression of the Tau Protein Gene after Ischemia

**Fig. 6:**
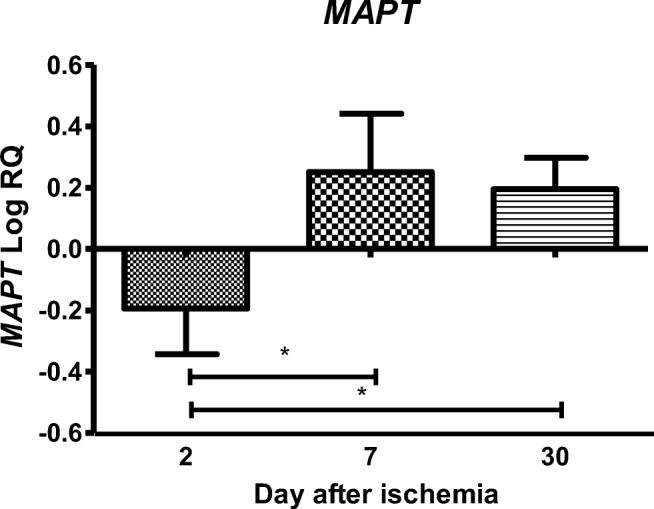
The mean gene expression levels of tau protein (*MAPT*) in the hippocampus CA3 area in rats 2 (*n* = 14), 7 (*n* = 8), and 30 (*n* = 8) days after 10-min global brain ischemia. Marked SD, standard deviation. Indicated statistically significant differences in levels of gene expression between 2 and 7 (*z* = 3.599, *p* = 0.0009) and between 2 and 30 days (*z* = 4.398, *p* = 0.00003) after ischemia (Kruskal-Wallis test). *p ≤ 0.001

In the CA3 region, the expression of the tau protein gene (*MAPT*) after ischemic damage with a survival of 2 days was lower than the control values and higher than the control values on days 7–30. On the second day after ischemia, the minimum was − 0.622-fold change and maximum − 0.001-fold change with median − 0.176-fold change. On the seventh day after ischemia, the minimum was 0.100-fold change and maximum 0.567-fold change with median 0.182-fold change. On the thirtieth day after ischemia, the minimum was 0.028-fold change and maximum 0.339-fold change with median 0.182-fold change. Figure 6 illustrates changes in the mean level of expression of the tau protein gene. The changes were statistically significant between 2 and 7 days and between 2 and 30 days after ischemia (Fig. 6).

## Discussion

In this study, we showed for the first time the time course of changes in gene expression: α- and β-secretase, presenilin 1 and 2, amyloid protein precursor, and tau protein associated with Alzheimer’s disease in the CA3 area of the hippocampus after 10-min brain ischemia in rats with 2, 7, and 30 days of survival. Our data provide the first known role of in vivo dysregulated selected genes related with Alzheimer’s disease in neuronal changes or death in the CA3 region of the hippocampus after ischemia-reperfusion injury. In addition, our data show that brain ischemia with recirculation activates neuronal changes and death in the CA3 region of the hippocampus in a manner dependent on amyloid and tau protein, thus determining a new and important way to regulate the survival and/or death of post-ischemic neurons. These data may support the theory that amyloid generated by ischemia and changes in the tau protein may be involved in the development of neuropathology of Alzheimer’s disease associated with ischemia [14, 15, 26, 27, 32–35].

In the present study, the expression of the α-secretase gene was below control values at all times studied. On the other hand, the expression of the β-secretase gene was below the control values 2–7 days after ischemia and the maximal increase in its expression was observed on day 30. Expression of the presenilin 1 gene was significantly elevated above the control values 2–7 days after ischemia and decreased below the control values at day 30 of recirculation. Expression of the presenilin 2 gene demonstrated an opposite trend to the expression of presenilin 1. Expression of the amyloid protein precursor gene was at all times above the control values after ischemia with a huge significant overexpression on day 7. Additionally, the expression of the tau protein gene was below the control values 2 days after ischemia, but the significant increase in its expression was observed on days 7–30. This study suggests amyloidogenic processing of the amyloid protein precursor in the CA3 region of the hippocampus, which is likely to end 30 days after the ischemic event, and this observation clearly contrasts with the amyloid generation in the CA1 region [14]. In the CA1 area, amyloid production was associated with the appearance of necrotic neuronal lesions on the second day after ischemia [36–39]. Membrane integrity appears to have been maintained in most CA3 pyramidal neurons during this time [11, 12]. Our results show that there is a discrepancy in time between the amyloidogenic processing of the amyloid protein precursor in the neurons of the CA3 and CA1 regions by approximately 28–30 days [14].

The evidence available to date indicates that at least three factors are indispensable for irreversible damage to the CA3 hippocampal area after ischemia during short-term survival (2–30 days), which is independent of both ischemia and reperfusion time. These include glutamate neurotoxicity, intraneuronal calcium loading [40], and tissue acidosis [9]. Overexpression of the amyloid protein precursor and dysregulation of both β- and γ-secretases induced by ischemia provides an excess of soluble form of amyloid [8, 9, 19, 32, 33, 36, 37], which in turn can act as an additional neurotoxin that interferes with neuronal function and increases neuronal death through multiple mechanisms, including additionally impaired calcium homeostasis [40], initiation of neuroinflammatory processes [41], loss of nervous system integrity [42, 43], and by affecting the permeability of the blood-brain barrier [9, 44–47]. Some evidence indicates that amyloid reduces neuroplasticity and contributes to increased neuronal susceptibility to ischemia [41, 48]. In addition, changes in the tau protein gene expression (7–30 days post-ischemia) trigger disturbances in neuronal microtubule transport, e.g., the amyloid protein precursor, and this observation clearly contrast with these types of changes in CA1 area on day 2 [15]. On the other hand, the overexpression of genes associated with Alzheimer’s disease during recirculation may be a self-sustaining *vicious cycle* that leads to progressive neurodegeneration beginning in CA1 and then passing to the CA3 region of the hippocampus [10, 16] and finally spreading to other brain regions. The pathological feature of the Alzheimer’s disease is the accumulation of amyloid plaques at first in the hippocampus and spreading gradually throughout the brain [9, 10, 41]. It is likely that the accumulation is caused by both increased generation and impaired amyloid clearance and/or the transfer of amyloid from the blood to the CA3 region of the hippocampus [49–51]. These data support previous immunohistochemical observations in both animals and humans after brain ischemia suggesting a direct relationship between ischemia and increased amyloidogenic processing of the amyloid protein precursor in the hippocampus [9, 36, 52–58]. These findings are consistent with previous studies in semi-quantitative analysis of secretases in local cerebral ischemia models and additionally confirm the participation of β-secretase [59] and presenilin 1 and 2 in ischemia-induced β-amyloid peptide generation [60, 61]. In total, upregulation of genes found in our study suggests that β-secretase (after 30 days), presenilin 1 (after 2–7 days) and 2 (after 30 days) are activated at different times after ischemia in the CA3 region of the hippocampus. These data indicate that in ischemic pyramidal neurons, the amyloid protein precursor, β-secretase, and γ-secretase interact to promote an additional pathological sequence that causes neuronal death. Activation of β-secretase may then trigger further apoptotic signaling by inducing caspase activation [28, 29] and DNA fragmentation. It should be emphasized that both events are also evident in the brains of Alzheimer’s disease patients [62]. In addition, the opposite trend in the expression of presenilin 1 and 2 indicates that two presenilins can play different roles under rat brain ischemia [21, 26]. Our model of brain ischemia confirmed that presenilin 2 probably played a role in the modulation of apoptosis [21, 28, 62], because neurons in the ischemic region of the CA3 began to be under the influence of generated amyloid and dysfunctional tau protein in 7–30 days after ischemia [21, 26]. Although presenilins seem to complement each other’s functions, presenilin 1 appears to provide a fundamental constitutive function, whereas presenilin 2 appears to act as an emergency aid in ischemic conditions [14, 21].

The presented results allow to understand progressive ischemic changes in the different areas of the hippocampus, delayed amyloid accumulation, and long-term ischemic pathogenesis of Alzheimer’s disease. Current data may partly help to explain the molecular mechanism of slower occurrence of neuronal damage and death in the area of the ischemic CA3 hippocampus than in CA1 [11, 12]. The above changes are associated with the atrophy of the hippocampus [8, 9, 63] and the development of dementia of the Alzheimer’s disease type, as it was previously observed [2, 32, 33, 64–66]. These results suggest that both aberrant gene expression associated with Alzheimer’s disease and the accumulation of their products due to ischemia-reperfusion brain injury may play a key role in the neurodegeneration of CA3 pyramidal neurons [32, 33, 36, 37, 47, 54–58, 67]. It also suggests that the above processes may be directly related to the death of neurons in the ischemic CA3 area. Our study shows that gene transcription associated with Alzheimer’s disease correlates positively with the state of ischemic neurons within 7–30 days after ischemia [8, 9, 11, 12]. Expression of the studied genes in the CA3 region did not decrease in late times (30 days) after ischemia, with the exception of presenilin 1 and α-secretase. This may be a reflection of the onset of the disappearance of pyramidal neurons in the CA3 region of the hippocampus [8, 9, 11, 12]. According to our data and the results of other authors, it can be concluded that focal and/or global brain ischemia with reperfusion interferes with the metabolism of the amyloid protein precursor and tau protein at both the gene and protein levels, and leads to amyloid accumulation and tau protein dysfunction [15, 23, 34, 36, 67–75]. The conclusions that can be drawn from the CA3 area study of genes in the ischemic hippocampus indicate that many genes contributing to neuronal death and the formation of amyloid, may be important for developing therapeutic targets in the treatment of dementia in Alzheimer’s disease. Therefore, of course, further research is needed on this topic. It seems that in our rat model of ischemic brain injury in vivo, neurodegenerative symptoms are less severe in CA3 compared with the CA1 area of hippocampus during 30 days of survival. Finally, damage to the CA3 area of the hippocampus is associated with irreversible memory impairment [2, 64–66]. Because memory impairment is the earliest symptom of Alzheimer’s disease, we have found that loss of neurons in the CA3 area of the hippocampus due to ischemia-reperfusion episode is sufficient and contributes to memory impairment in a manner dependent on amyloid and tau protein at very early stages after brain ischemia and Alzheimer’s disease.

It is noteworthy that the hippocampus may also play an important role in other neurological conditions, such as epilepsy, which often accompanies Alzheimer’s disease. For example, during the induction of kindled seizures in rats, the increased convulsive response on day 25 was clearly associated with increased expression of a number of plasticity-regulating proteins [76]. It has also been documented that the kindling caused a huge increase in the number of neuronal cells possessing doublecortin (migration marker) in the dorsal hippocampus [77]. Acute seizures, 1 h after a single injection of pentylenetetrazol in rats, induced a hippocampal response reflected in a significant increase in c-fos mRNA [78]. Finally, after cardiac arrest in rats that caused cerebral ischemia, spontaneous seizure activity was observed in 68% of rats, and these animals were then susceptible to the induction of audiogenic seizures [79]. In the light of the presented data, the hippocampus probably takes part in these phenomena.

## Conclusion

Our research indicates the key role of brain ischemia in the development of Alzheimer’s disease through primary neurodegeneration of various hippocampal regions, including CA3 area, and cognitive decline that progress with prolonged reperfusion times. Thus, bilateral damage to the abovementioned region results in the deterioration of short-term memory, which leads to the inability to create new memories. Changes in the CA3 region in the brain after hippocampal ischemia are particularly important in memory impairment, including Alzheimer’s disease. However, further research is needed to determine whether the damage and death of pyramidal neurons in the CA3 region are causative events or independent consequences of ischemia occurring in parallel and leading to the development of post-ischemic dementia. Apparently, prevention of brain ischemia and early treatment of ischemic stroke may have important implications for the development of Alzheimer’s disease and merit further research. Finally, the rat model used in this study seems to be a useful experimental approach to determining the role of genes associated with Alzheimer’s disease. Through in-depth research into the common genetic mechanism associated with these two neurological disorders, our findings can accelerate the current understanding of neurobiology in Alzheimer’s disease and cerebral ischemia, and lead future research into Alzheimer’s disease or cerebral ischemia in new directions.
